# Stochastic Endogenous Replication Stress Causes ATR-Triggered Fluctuations in CDK2 Activity that Dynamically Adjust Global DNA Synthesis Rates

**DOI:** 10.1016/j.cels.2018.05.011

**Published:** 2018-06-13

**Authors:** Leighton H. Daigh, Chad Liu, Mingyu Chung, Karlene A. Cimprich, Tobias Meyer

**Affiliations:** 1 Department of Chemical and Systems Biology, Stanford University School of Medicine, Stanford, CA 94305, USA; 2 These authors contributed equally; 3 Lead Contact

## Abstract

Faithful DNA replication is challenged by stalling of replication forks during S phase. Replication stress is further increased in cancer cells or in response to genotoxic insults. Using live single-cell image analysis, we found that CDK2 activity fluctuates throughout an unperturbed S phase. We show that CDK2 fluctuations result from transient ATR signals triggered by stochastic replication stress events. In turn, fluctuating endogenous CDK2 activity causes corresponding decreases and increases in DNA synthesis rates, linking changes in stochastic replication stress to fluctuating global DNA replication rates throughout S phase. Moreover, cells that reenter the cell cycle after mitogen stimulation have increased CDK2 fluctuations and prolonged S phase resulting from increased replication stress-induced CDK2 suppression. Thus, our study reveals a dynamic control principle for DNA replication whereby CDK2 activity is suppressed and fluctuates throughout S phase to continually adjust global DNA synthesis rates in response to recurring stochastic replication stress events.

## In Brief

Live single-cell microscopy reveals a control principal that helps maintain proper duplication of genetic material. Upon inevitable DNA replication stress during S phase, cells signal through ATR to attenuate CDK2 activity, which then decreases global DNA synthesis rate. This feedback enables dynamic modulation of S-phase progression.

## Graphical Abstract

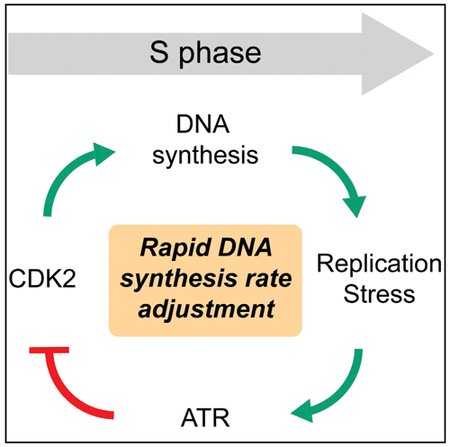

## INTRODUCTION

Cells must survey and maintain genomic integrity to ensure proper cellular function and faithful duplication and distribution of DNA to daughter cells. Genomic integrity is constantly challenged by endogenous and exogenous threats. This is especially true during S phase, when stalling of DNA replication forks places the genome at increased risk for DNA damage and ensuing mutations. Sources of endogenous replication stress that lead to DNA damage include transcription-replication conflicts, complicated DNA secondary structures, and resource limitation (Cortez, 2015). Here, we use a previously described definition of replication stress as any process that impairs DNA synthesis and/or replication fork progression (Saldivar et al., 2017). Replication stress and DNA damage during S phase are driving forces in the development of cancer and aging, possibly due to the effects of replication stress and subsequent mutations on tissue stem cells as they re-enter the cell cycle from quiescence (Magdalou et al., 2014). Stochastic damage events that occur during DNA replication have been proposed to be the predominant source of cancer-causing mutations (Tomasetti et al., 2017; Tomasetti and Vogelstein, 2015). Thus, understanding DNA damage and replication stress signaling in unperturbed cells is of fundamental importance to understanding the origin of cancer-causing mutations (Gaillard et al., 2015).

**Figure 1.**
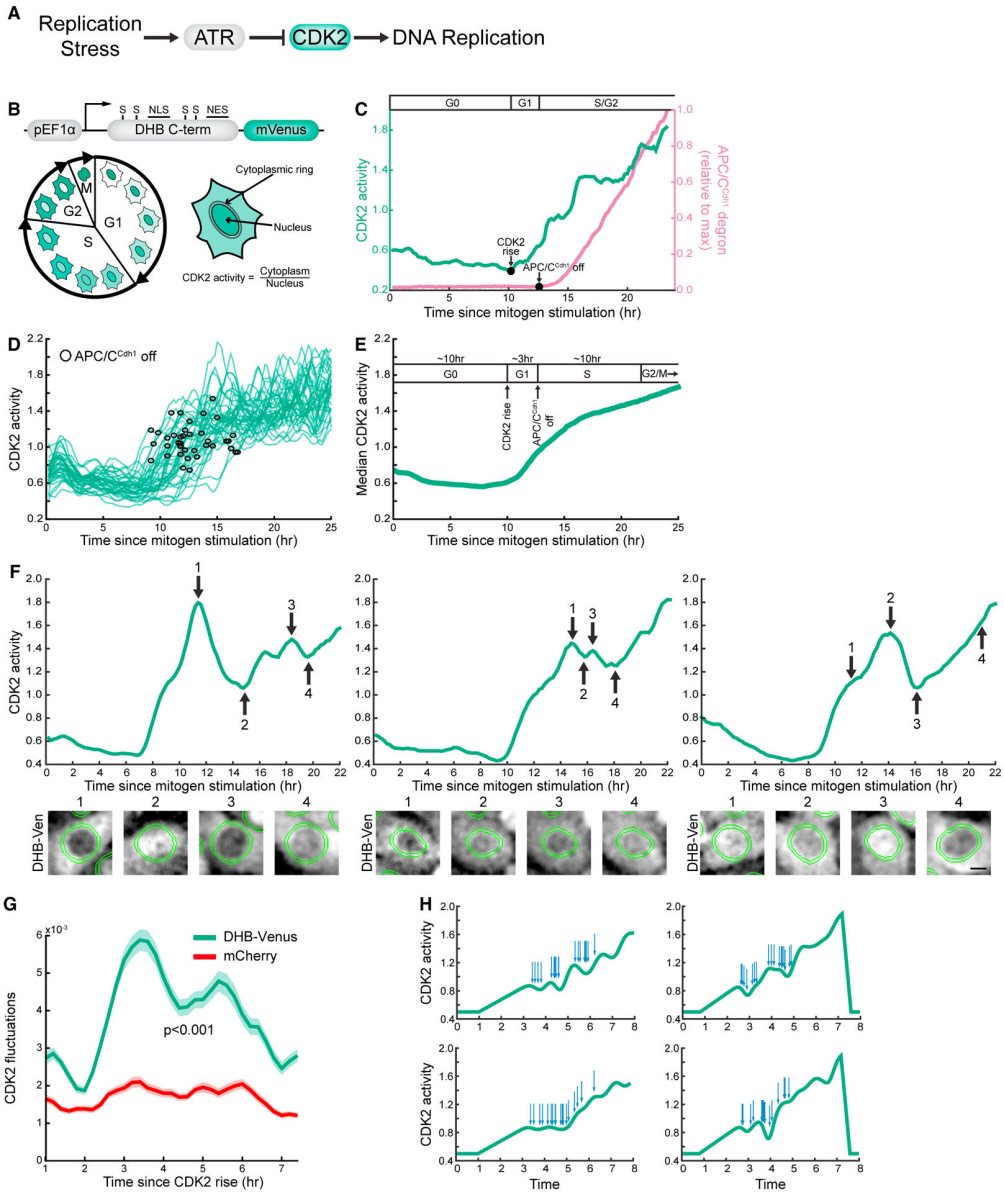
Single-Cell Analysis Reveals Heterogeneous, Fluctuating CDK2 Activity in S Phase (A) Simplified model showing that activation of ATR signaling by replication stress results in CDK2 inhibition. CDK2 activity is important for many facets of S-phase progression, including the firing of new DNA replication origins. (B) Design of the CDK2 reporter characterized in Spencer et al. (2013). C-terminal fragment of DNA helicase B (DHB) fused to mVenus. Nuclear export signal (NES), nuclear localization signal (NLS), and serine residues phosphorylated by CDK2 are indicated. Upon phosphorylation by CDK2, the reporter translocates from the nucleus to the cytoplasm. CDK2 activity can be recorded as the ratio of cytoplasmic to nuclear fluorescence intensity in live cells. (C) Representative single-cell trace showing live-cell measurement of CDK2 activity and APC/C^Cdh1^ degron levels. Time of CDK2 activity increase (CDK2 rise) and APC/C^Cdh1^ inactivation are indicated. The initial rise in CDK2 activity was used to determine the transition from G0 to G1 phase. The accumulation of a fluorescently tagged anaphase-promoting complex substrate, geminin, was used to determine the time of APC/C^Cdh1^ inactivation, which corresponds to S-phase entry. (D) Single-cell CDK2 activity traces of 40 cells exiting quiescence upon mitogen stimulation. The time point of APC/C^Cdh1^ inactivation is indicated on each trace with an open black circle. Computational gating was performed for cells with initially low CDK2 activity that increased over the course of imaging. One of n = 2 biological replicates. (E) Median trace of cells that increase CDK2 activity coming out of quiescence (n > 1,000). Approximate times for each phase of the cell cycle in MCF10A cells exiting quiescence following mitogen stimulation is indicated above the averaged trace. Times are based on data presented here and in previous studies (Spencer et al., 2013; Cappell et al., 2016). One of n = 2 biological replicates. (F) Representative single-cell CDK2 activity traces of cells exiting quiescence showing fluctuations in CDK2 activity. Raw images of the DHB-Ven construct for the indicated time points are shown beneath each trace. The cytoplasmic ring mask used for calculating the cytoplasmic signal is shown in green. Scale bar: 5 μm. (G) Measurement of CDK2 fluctuations in single cells expressing the DHB-Ven CDK2 reporter and an untagged mCherry control following release from quiescence. Cells were computationally aligned to the time of initial CDK2 activity rise. Fluctuations were quantified by determining the difference squared between individual traces and a best-fit curve. Lines indicate mean trace and shaded regions indicate SEM (n = 590 cells). One of n = 2 biological replicates. (H) Computational model of linearly increasing CDK2 activity traces. Twenty stress events (indicated by arrows above CDK2 trace) were simulated at random time points throughout the length of S phase. Each damage event had an equivalent inhibitory effect on CDK2 activity. See also Figures S1 and S2.

Two signaling pathways are particularly important for the maintenance of genome integrity. First, DNA double-strand breaks are detected by ataxia-telangiectasia mutated (ATM), which initiates a DNA damage checkpoint and cell-cycle arrest (Bensimon et al., 2011). Mice lacking ATM exhibit growth retardation, early lymphomas, and pleiotropic phenotypes associated with human ATM deficiency (Barlow et al., 1996). Second, the master regulator of the replication stress response is ataxia-telangiectasia and RAD3-related (ATR) kinase (Saldivar et al., 2017). ATR is recruited to RPA-coated single-stranded DNA and initiates a signaling cascade in part via activation of the downstream kinase, Chk1 (Berti and Vindigni, 2016). When ATR is experimentally inhibited via small interfering RNA knockdown or small molecule inhibition, cells undergo extensive replication stress and DNA damage due to unrestrained origin firing (Beck et al., 2010; Syljuasen et al., 2005; Toledo et al., 2011). This argues that ATR stress signaling is critical to prevent DNA damage during a normal unperturbed S phase. ATR and ATM activation resolve replication stress in part by inhibiting cell-cycle progression via CDC25A inhibition, which allows Wee1 to inhibit cyclin-dependent kinases (CDKs). CDK2 activity drives S-phase progression by many mechanisms (Pines, 1999), including the promotion of DNA replication origin firing (Beck et al., 2012; Petermann et al., 2010). While molecular links from replication stress and DNA damage to CDK2, and from CDK2 to DNA replication, are well established (Figure 1A), ATR and ATM have additional CDK2-independent regulatory roles to stabilize replication forks, resolve stalled replication forks, and repair damage (Bensimon et al., 2011; Saldivar et al., 2017). Furthermore, CDK2 activity and DNA replication rates have been shown to be controlled by a number of other regulatory processes (Burgess and Misteli, 2015; Saldivar et al., 2017; Tubbs and Nussenzweig, 2017). Due to the multitude of regulatory mechanisms, it is not yet known whether normally occurring replication stress and ATR signaling in S phase cause global fluctuations in CDK2 activity. It is also not known whether global fluctuations in CDK2 activity, if they indeed exist in unperturbed cells, cause corresponding global slowing and acceleration of the rate at which new origins are fired and S phase progresses in an individual cell.

Previous studies of cell-cycle progression and replication stress have predominantly relied on bulk-cell assays and population-level analyses that can obscure stochastic and fluctuating signaling events. The use of single-cell analysis enables the detection of intercellular variability and the ability to study inherently heterogeneous phenomena that affect the cell cycle (Matson and Cook, 2017). Moreover, while recent studies used live-cell measurements to monitor p53 and other DNA damage signaling effectors (Arora et al., 2017; Barr et al., 2017; Paek et al., 2016; Stewart-Ornstein and Lahav, 2017), the study of the immediate response to replication stress and DNA damage has mostly been limited to fixed-cell analysis. The recent availability of fluorescent reporters to monitor CDK2 activity and cell-cycle phases (Sakaue-Sawano et al., 2008; Spencer et al., 2013) now makes it possible to understand how CDK2 activity, DNA replication rates, and S-phase progression speed are dynamically regulated by normally occurring replication stress and DNA damage in unperturbed cells.

Here, we employ live-cell imaging and single-cell analysis to study the impact of endogenous replication stress on CDK2 activity and S-phase progression. We show that while, on average, CDK2 activity steadily increases throughout S phase, individual cells exhibit great heterogeneity with marked fluctuations in CDK2 activity. Furthermore, CDK2 activity decreases rapidly in response to replication stress by ATR-mediated inhibition, and the degree of CDK2 inhibition is proportional to the extent of replication stress encountered. Strikingly, repression of CDK2 activity does not require any exogenous stressors, explaining how stochastic endogenous replication stress can produce ATR-mediated fluctuating CDK2 activity in individual cells. We further show that the fluctuations in CDK2 activity dynamically control parallel decreases and increases of the global rate of DNA synthesis. Finally, we demonstrate that the first S phase in quiescent cells stimulated with mitogens exhibits a unique CDK2 activity pattern with more CDK2 fluctuations and a longer S phase that is distinct from continuously cycling cells. We were able to attribute the increase in CDK2 activity fluctuations to increased DNA damage and replication stress that quiescent cells encounter when they re-enter the cell cycle. Taken together, our results reveal a control principle whereby stochastic replication stress events dynamically adjust the global DNA replication rate and speed of S-phase progression by triggering rapid ATR-mediated global repressions in CDK2 activity.

## RESULTS

### Single-Cell Analysis Reveals Heterogeneous, Fluctuating CDK2 Activity in S Phase

To better understand how replication stress regulates CDK2 activity and S-phase progression, we monitored dynamic changes in CDK2 activity during an unperturbed S phase. We have previously shown that CDK2 activity can be monitored in individual cells by measuring the subcellular localization of a fragment of DNA helicase B tagged with the fluorescent protein mVenus (DHB-Ven) (Spencer et al., 2013). CDK2 activity can thus be measured in live cells by comparing the cytoplasmic (CDK2-phosphorylated) to nuclear (unphosphorylated) ratio of DHB-Ven (Figure 1B). More precisely, the CDK2 reporter monitors in G1 and early S phase Cyclin E/CDK2 activity and later in S and G2 also Cyclin A/CDK2 and Cyclin A/CDK1 activities (Cappell et al., 2016; Schwarz et al., 2018; Spencer et al., 2013). Moreover, we and others have shown that S-phase entry and the initiation of DNA synthesis can be precisely determined in live cells by measuring the accumulation of a truncated, fluorescently tagged version of the anaphase-promoting complex (APC/C^Cdh1^) substrate, Geminin (Gem-mChy) (Figure 1C) (Cappell et al., 2016; Sakaue-Sawano et al., 2008). To measure changes in CDK2 activity in S phase in single cells, we performed time-lapse microscopy on the non-transformed breast epithelial cell line, MCF10A, expressing DHB-Ven and Gem-mChy. In MCF10A cells, the measured activity is mostly from CDK2, as addition of a CDK1 inhibitor caused only a small reduction in measured reporter activity in all phases of the cell cycle (Figure S1) (Spencer et al., 2013).

Similar to previous results, CDK2 activity is initially low as cells exit quiescence before beginning to rise approximately 10 hr after mitogen stimulation (Figures 1D and 1E) (Spencer et al., 2013). S-phase entry, marked by APC/C^Cdh1^ inactivation and Gem-mChy accumulation, occurs on average 3 hr after the initial rise in CDK2 activity (Figures 1D and 1E). CDK2 activity continues to build up throughout S and G2 phase, showing a smooth progression when many traces are averaged together (Figure 1E). However, examination of single-cell CDK2 activity traces reveals that CDK2 activity does not uniformly increase during S phase (Figure 1D). Rather, CDK2 activity shows marked fluctuations throughout S phase, with activity decreases and plateaus frequently observed (Figure 1F, Video S1).

To quantitatively assess the degree and timing of CDK2 fluctuations, we calculated the deviation of individual CDK2 activity traces from a best fit of a third-degree polynomial for each trace (Figure S2A; STAR Methods). This analysis revealed that detected fluctuations increase greatly upon S-phase entry, approximately 3 hr after CDK2 rise (Figure 1G). To confirm that these fluctuations represent true CDK2 volatility and not measurement noise, we performed the same analysis in cells expressing the DHB-Ven CDK2 sensor and an unconjugated mCherry fluorescent protein that was more evenly distributed between the nucleus and cytoplasm. We found that the DHB-Ven fluctuations increase significantly above background during S phase (Figures 1G and S2A), demonstrating that our measurement of fluctuations in S-phase CDK2 activity is not significantly affected by errors in tracking or analysis. This was also true when we only selected cells with an average mCherry cytoplasmic:nuclear ratio greater than 1.1 in the analysis (Figures S2B and S2C). We further confirmed our results by quantifying CDK2 fluctuations using an independent method based on analysis of the second derivative of each trace. Similarly, we observed that CDK2 fluctuations increase above background 3 hr after CDK2 rise, at the approximate time of S-phase entry (Figure S2D). Due to limitations in the ability to precisely determine the time point of the S/G2 transition and increased measurement noise at high cytoplasmic:nuclear ratios, we decided to focus our investigations on CDK2 fluctuations in early to mid S phase (CDK2 activity <1.3), where our measurements most accurately reflect S-phase CDK2 activity. Thus, these single-cell measurements reveal that heterogeneous, stochastic fluctuations in CDK2 activity occur during an unperturbed S phase.

We hypothesized that the fluctuating CDK2 activity time courses are generated by stochastic DNA damage or replication stress events during S phase. To test whether such a mechanism can in principle generate fluctuations, we modeled a system where CDK2 activity is linearly increasing during S phase of a cell cycle. We then used Monte Carlo simulations to generate in different runs 20 stress events at random times during S phase that transiently suppress CDK2 activity (Figure 1H, STAR Methods). We observed that the uneven random distribution of individual stresses often generates cluster-like events that result in fluctuating CDK2 activity, resembling the measured CDK2 activity fluctuations in live single cells (Figures 1C and 1F). As a control, such stochastic fluctuations in the simulated CDK2 activity traces were accurately detected with our polynomial fit method (Figure S2E). This demonstrates that a limited number of stochastic events that transiently repress CDK2 activity can in principle generate the observed fluctuations in CDK2 activity.

### CDK2 Activity Is Transiently Suppressed by Exogenous Replication Stress

**Figure 2.**
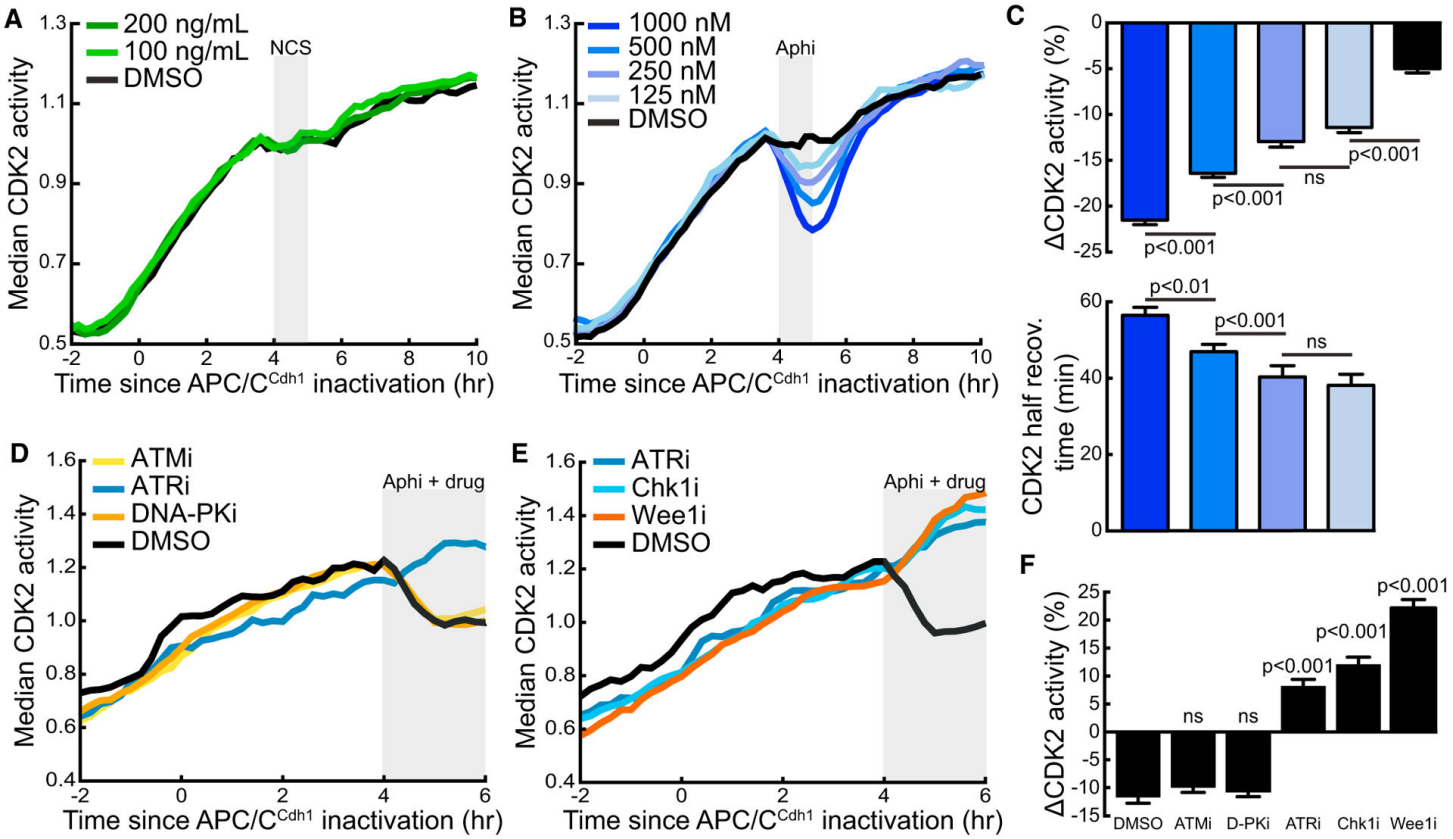
CDK2 Activity Is Transiently Suppressed by Exogenous Replication Stress (A) Median CDK2 traces of cycling MCF10A cells computationally gated for cells in S phase during 1-hr neocarzinostatin (NCS) pulse (n = 250, 200 ng/mL; n = 246, 100 ng/mL; n = 235, DMSO). Gray box indicates the time of NCS pulse. One of n = 2 biological replicates. (B) Median CDK2 traces of cycling MCF10A cells computationally gated for cells in S phase at the time of aphidicolin 1-hr pulse at the indicated concentrations (n = 300, 1000 nM; n = 336, 500 nM; n = 283, 250 nM; n = 221, 125 nM; n = 260, DMSO). Gray box indicates the time of aphidicolin pulse. One of n = 2 biological replicates. (C) The percentage change in CDK2 activity measured from the time frame when aphidicolin was added to the time of aphidicolin washout. CDK2 recovery time was calculated as the time after aphidicolin washout for CDK2 activity to recover to half of the level of CDK2 activity in the time point immediately preceding aphidicolin addition. Measurements are single-cell analysis of cells in (B). Mean values ± SEM are shown. One of n = 2 biological replicates. ns, not significant. (D and E) Median traces of cycling cells computationally gated for cells in S phase during treatment with aphidicolin and the indicated DDR inhibitor (n = 231, ATMi; n = 149, ATRi; n = 254, DNA-PKi; n = 186, Chk1i; n = 158, Wee1i; n = 146, DMSO). Gray box indicates time of drug addition. One of n = 2 biological replicates.. (F) Percentage change from (D) and (E) in CDK2 activity of individual cells treated with aphidicolin and the indicated DDR inhibitor. Percentage change was calculated as the change in CDK2 activity from the frame before drugs were added to the frame at 1 hr following drug addition. Mean values ± SEM are shown. Significance values are reported for each condition compared with DMSO control. One of n = 2 biological replicates. See also Figure S3.

We next investigated known stress signaling pathways that may regulate CDK2 activity fluctuations in S phase. We first tested the hypothesis that CDK2 fluctuations occur in response to DNA strand breaks by treating cells with the neocarzinostatin (NCS), which generates DNA double-stranded breaks via free radical production. Surprisingly, there was no immediately apparent effect of NCS treatment on S-phase CDK2 activity (Figure 2A) despite significant detectable DNA damage at later time points (Figure S3). Given CDK2’s position as a terminal phosphorylation target of the ATR-Chk1-Cdc25a/Wee1 pathway, we reasoned that S-phase fluctuations in CDK2 activity might instead represent the cellular response to replication stress and not DNA damage events that may occur independently of replication stress. We treated cells with various doses of aphidicolin, which stalls replication fork progression via direct inhibition of DNA polymerase, to exogenously induce replication stress. Following aphidicolin addition, we observed an immediate, rapid decrease in the CDK2 activity of S-phase cells (Figure 2B). The decrease in CDK2 activity closely correlated with the dose of aphidicolin, demonstrating that inhibition of CDK2 activity is proportional to the degree of replication stress (Figure 2C). Markedly, CDK2 activity increased with a half-life of approximately 40 min upon washout of low doses of aphidicolin, demonstrating a rapid recovery of CDK2 activity after the removal of a replication stress-inducing stimulus. The length of time required for CDK2 activity to return to pre-treatment levels increased from 40 to 60 min with increasing aphidicolin dose, showing fast responses with a small correlation between CDK2 recovery time and the extent of replication stress (Figure 2C).

To determine which DNA damage response (DDR) pathway is mostly responsible for transient CDK2 inhibition, we combined aphidicolin treatment with small molecule inhibition of the major DNA damage-responsive kinases, ATR, ATM, and DNA-PK. Inhibition of ATM or DNA-PK had no significant effect on the CDK2 response to aphidicolin treatment (Figure 2D). However, ATR inhibition abrogated the CDK2 response to aphidicolin and allowed CDK2 activity to continue to increase despite aphidicolin-induced replication stress. Moreover, inhibition of kinases downstream of ATR signaling, Chk1 and Wee1, also prevented a CDK2 response to aphidicolin (Figure 2E). Quantification of the CDK2 response to aphidicolin treatment in the presence of the various DDR inhibitors revealed no difference between ATM or DNA-PK inhibition and DMSO, while inhibition of ATR, Chk1, or Wee1 allowed CDK2 activity to continue to increase in the presence of aphidicolin (Figure 2F). Replication stress-induced CDK2 inhibition during S phase therefore occurs primarily through the ATR pathway, as concluded by previous studies using bulk-cell biochemical approaches (Cimprich and Cortez, 2008). Taken together, these results demonstrate that a brief period of replication stress triggers a rapid ATR-mediated 40-min-long repression of CDK2 activity.

### Stochastic Endogenous Replication Stress Events Generate Fluctuating CDK2 Activity

**Figure 3.**
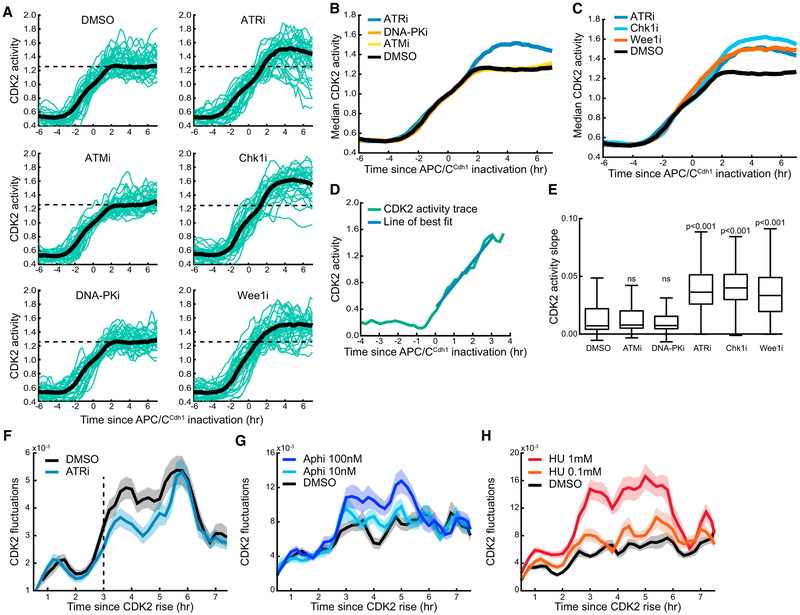
Stochastic Endogenous Replication Stress Events Generate Fluctuating CDK2 Activity (A)Thirty random single-cell traces (green lines) and median trace (black line) of cells aligned to time of APC/C^Cdh1^ inactivation following release from quiescence. The indicated inhibitors or DMSO were added 8 hr following mitogen stimulation, prior to APC/C^Cdh1^ inactivation. Median traces were calculated from the entire cell population (n = 583, DMSO; n = 757, ATMi; n = 673, DNA-PKi; n = 611, ATRi; n = 606, Chk1i; n = 448, Wee1i). Dashed line indicates approximate CDK2 activity plateau determined in the DMSO condition. Cells were computationally gated for cells that increase CDK2 activity out of quiescence. (B and C) Median traces from (A) are shown on the same graph. One of n = 2 biological replicates. (D) Example of single-cell analysis of CDK2 S-phase dynamics by drawing a line of best fit (blue line) from the time of S-phase entry to the time point when CDK2 activity (green line) crosses a threshold value of 1.5 and calculating the slope of the best-fit line. (E) Boxplots of single-cell analysis of S-phase CDK2 activity slope in cells exiting quiescence from (A). Significance values are reported for each condition compared with DMSO control. One of n = 2 biological replicates. (F) Fluctuation analysis of CDK2 activity in cells treated with DMSO or an ATR inhibitor prior to S phase and computationally aligned to the time of CDK2 activity rise. Lines indicate mean and shaded regions indicate SEM (n = 220 for both conditions). One of n = 2 biological replicates. (G and H) Fluctuation analysis of CDK2 activity in cells treated with indicated doses of hydroxyurea (HU) or aphidicolin (aphi) and computationally aligned to the time of CDK2 activity rise. Gating was performed to only include cells in late G1/early S phase (−1 to 4 hr relative to APC/C^Cdh1^ inactivation) at the time of drug addition. Lines indicate mean and shaded regions indicate SEM (n = 350 for all conditions). One of n = 2 biological replicates. See also Figure S4.

We next tested whether ATR signaling also influences CDK2 activity in individual, unperturbed cells. Even in the absence of exogenous replication stress, cells experience replication fork stalling due to a variety of endogenous causes, including an insufficient pool of deoxynucleotide triphosphates (dNTPs), DNA lesions, and collisions with transcription complexes (Zeman and Cimprich, 2014). Consistent with a role for ATR activation by endogenous sources of replication stress, ATR inhibition resulted in an accelerated increase in CDK2 activity during S phase (Figures 3A and 3B). Inhibition of Chk1 and Wee1 also resulted in a more rapid increase in S-phase CDK2 activity, similar to ATR inhibition (Figures 3A and 3C). This increase in CDK2 activity in response to Wee1 inhibition may explain the previous observation that Wee1 inhibitors shorten the time to mitosis (De Witt Hamer et al., 2011; Wang et al., 2004). No increase of S-phase CDK2 activity was observed following ATM or DNA-PK inhibition (Figures 3A and 3B). Notably, Wee1 inhibition had no effect on the ramp-up of CDK2 activity in G1 phase and started to increase CDK2 activity only after the start of S phase, arguing that ATR, Chk1, and other damage pathways that regulate CDC25/Wee1 do not significantly restrict CDK2 signaling during G1 phase in unperturbed cells.

To quantify the effects of the respective inhibitors, we calculated the rate of CDK2 activity increase during S phase in single cells by measuring the slope of a best-fit line to each individual trace beginning at the time of S-phase entry (Figure 3D). Inhibition of ATR resulted in a greater slope of CDK2 activity compared with DMSO, indicating that CDK2 activity increases more rapidly in S phase when the ATR-mediated replication stress response is inhibited (Figure 3E). Inhibition of Chk1 and Wee1 gave similar results to ATR inhibition, while inhibition of ATM or DNA-PK had no effect on the slope. These results show that the ATR pathway controls the rate at which CDK2 activity increases during S phase in unperturbed cells. Furthermore, inhibition of ATR decreases CDK2 fluctuations in single cells compared with DMSO treatment (Figure S4A), particularly in early S phase (Figure 3F). Similar results were observed upon inhibition of Chk1 or Wee1 (Figure S4B). DNA-PK inhibition had little effect on S-phase CDK2 fluctuations, while ATM inhibition slightly decreased CDK2 fluctuations (Figure S4C).

To further validate the contribution of replication stress to CDK2 fluctuations, we treated cells with low doses of aphidicolin to induce mild replication stress and observed an increase in the observed fluctuations (Figure 3G). Treating cells with hydroxyurea, which induces replication stress via dNTP depletion, similarly caused an increase in CDK2 fluctuations (Figure 3H). Overall, these results support a model that stochastic replication stress events produce fluctuating CDK2 activity in single cells by ATR-mediated recurring transient repression of CDK2 activity.

### Endogenous Fluctuations in CDK2 Activity Dynamically Decrease and Increase Global DNA Synthesis Rate

We next determined whether normally occurring fluctuations in CDK2 activity in unperturbed cells could affect the rate of DNA replication. Previous studies showed that CDK2 activity is required for the firing of new replication origins during S phase (Beck et al., 2012; Petermann et al., 2010) and can regulate the efficiency of origin firing in *Xenopus* egg extracts (Krasinska et al., 2008). ATR activity also influences origin firing and DNA replication through mechanisms separate from CDK2 activity regulation, including inhibition of helicase activation, stabilizing replication forks, and inhibition of CDK activity (Saldivar et al., 2017).

**Figure 4.**
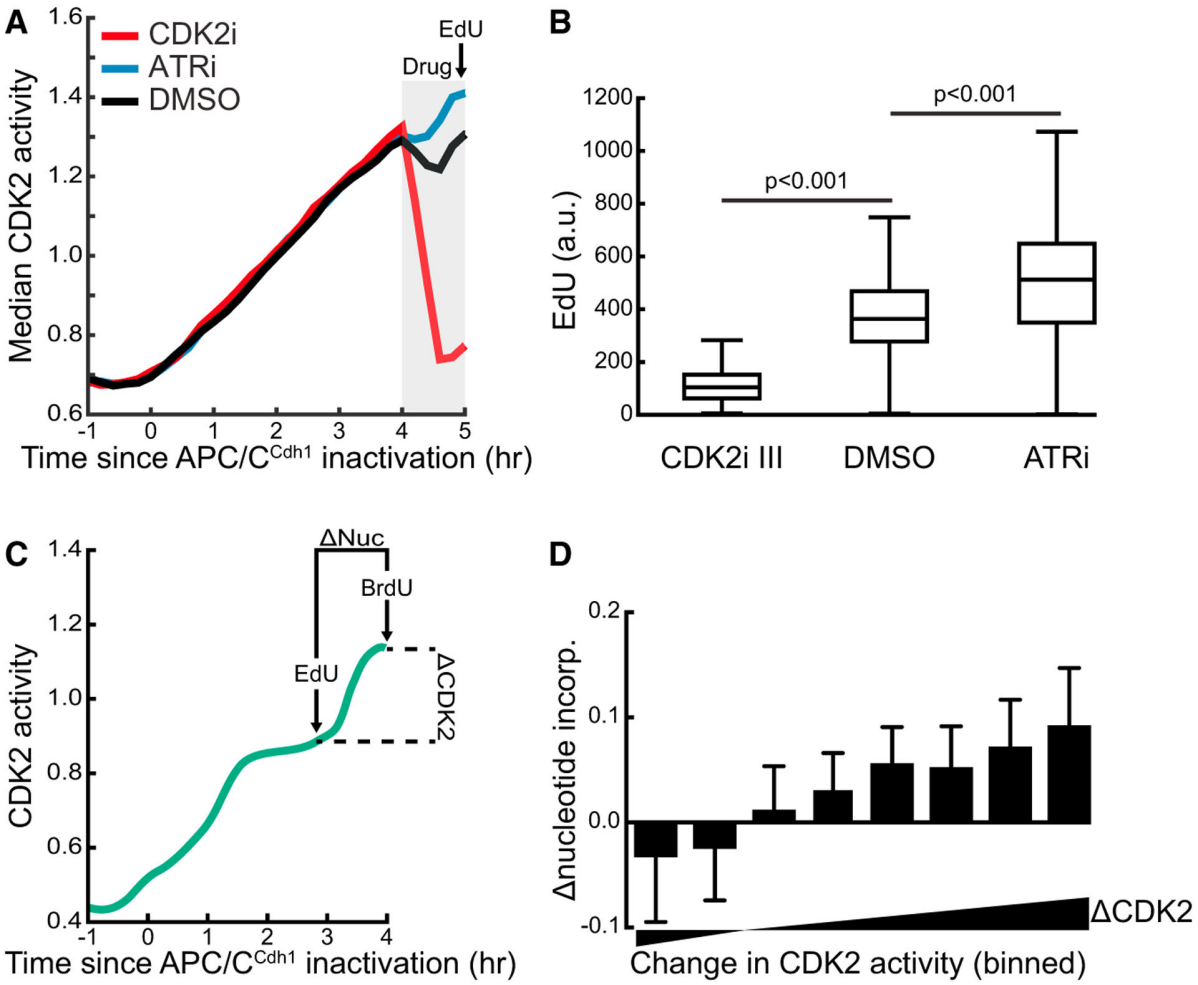
Endogenous Fluctuations in CDK2 Activity Dynamically Decrease and Increase Global DNA Synthesis Rate (A) Median traces of cycling cells gated for cells in S phase when treated with indicated drugs (n > 500 for each condition). One of n = 2 biological replicates. (B) Boxplot of EdU values in individual cells from (A) treated with the indicated drugs. (C) Experimental design for co-labeling of cells with EdU and BrdU to compare DNA synthesis rate at different points in time in individual cells. Changes in CDK2 activity and changes in nucleotide incorporation rate were analyzed in individual S-phase cells. (D) Cycling cells in S phase at the time of nucleotide addition were binned based on the change in CDK2 activity from EdU pulse to BrdU pulse and median Dnucleotide values ± SEM were calculated for each bin (n > 70 cells for each bin). Bins range from −0.10 to 0.25 with a step size of 0.05. By linear regression, r^2^ = 0.96. One of n = 2 biological replicates. incorp., incorporation.

We tested the hypothesis that fluctuating CDK2 activity during S phase could serve as a mechanism by which cells dynamically control the global rate of DNA synthesis. Such a mechanism would allow cells to integrate the current overall replication stress and adjust the overall DNA replication rate accordingly. This feedback control could be achieved by a CDK2-dependent transient and global reduction in the firing of new origins under conditions of high replication stress until the source of the stress is resolved. To confirm a role of CDK2 activity in regulating the rate of DNA replication, we added a CDK2 inhibitor to induce a rapid decrease in CDK2 activity or an ATR inhibitor to prevent ATR-mediated regulation of DNA replication (Figure 4A). Indeed, 5-ethynyl-2′-deoxyuridine (EdU) incorporation decreased following CDK2 inhibition, while ATR inhibition resulted in an increased nucleotide incorporation rate (Figure 4B).

Having confirmed that the level of CDK2 activity can control the global rate of DNA synthesis, we next determined if the magnitude of the endogenous, naturally occurring fluctuations in CDK2 activity are correlated with corresponding changes in the rate of DNA synthesis. To do this, unperturbed cells were pulsed with EdU for 10 min, washed, and incubated for 50 min with no nucleotide analogs present, then pulsed with bromodeoxyuridine (BrdU) for 10 min prior to fixation and visualization (Figure 4C). We were then able to compare changes in naturally occurring CDK2 activity with changes in the relative rate of nucleotide incorporation at these two separated time windows. After binning cells based on the changes in CDK2 activity recorded, we observed that cells with the greatest increase in CDK2 activity also had the greatest increase in DNA synthesis rate over the 1-hr time period, while decreasing bins of CDK2 activity changes correlated with lower relative rates of nucleotide incorporation (Figure 4D). Therefore, CDK2 activity regulates the global rate of DNA replication, and normal endogenous fluctuations in CDK2 activity are paralleled by changes in the global DNA synthesis rate.

### Increased Replication Stress in Mitogen-Induced Exit from Quiescence Causes Increased ATR-Mediated Fluctuations of CDK2 Activity and a Prolonged S Phase

**Figure 5.**
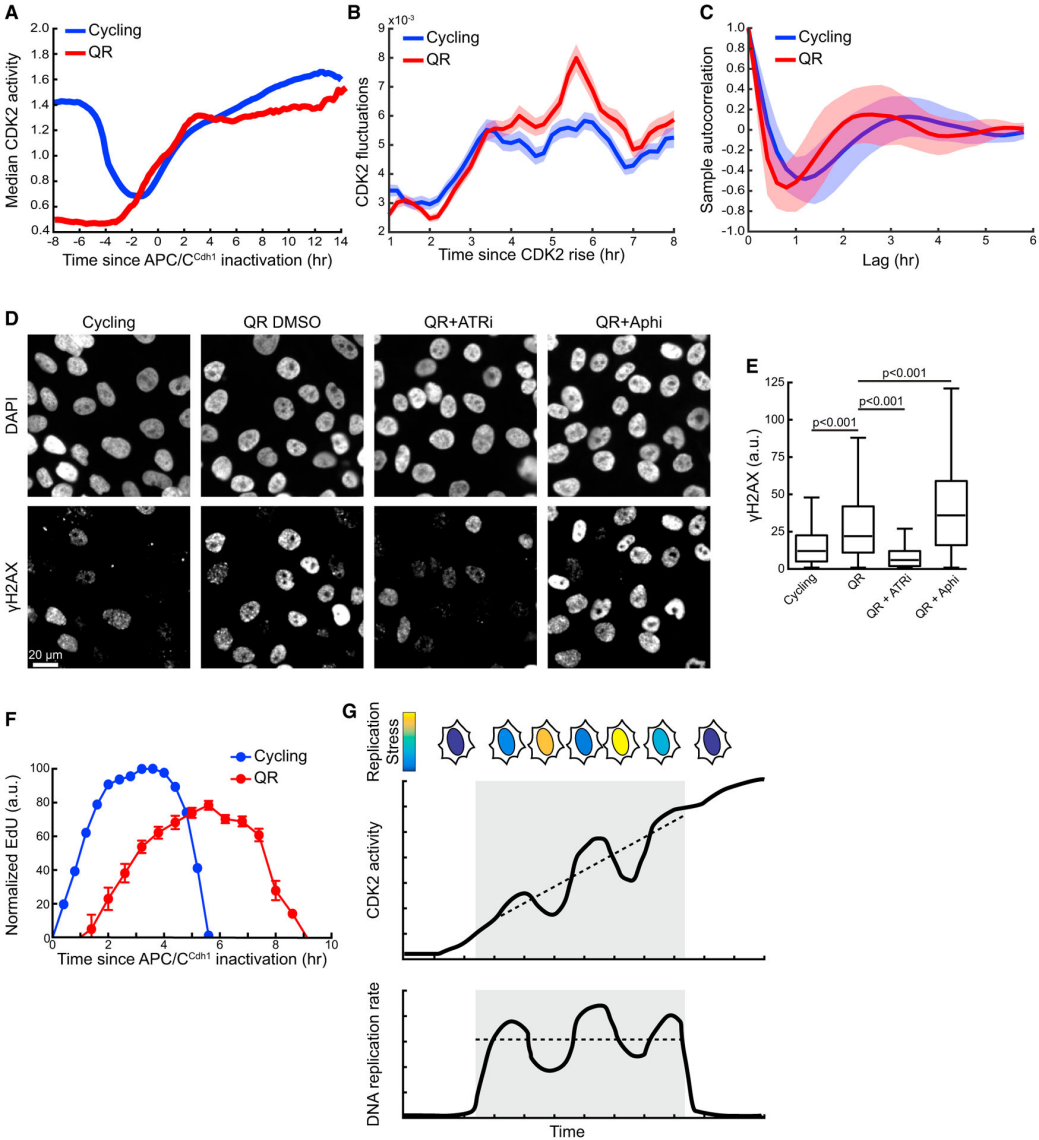
Increased Replication Stress in Mitogen-Induced Exit from Quiescence Causes Increased ATR-Mediated Fluctuations of CDK2 Activity and a Prolonged S Phase (A) Median CDK2 activity traces aligned to time of APC/C^Cdh1^ inactivation for continuously cycling cells and quiescence-release (QR) cells stimulated with mitogens (n = 300 cells for each condition). One of n = 2 biological replicates. (B) Fluctuation analysis of CDK2 activity in S phase of continuously cycling cells and QR (n = 1,000 cells for each condition). Lines indicate mean and shaded regions indicate SEM. One of n = 2 biological replicates. (C) Autocorrelation analysis of S-phase CDK2 activity in cycling and QR cells (n = 300 cells for each condition). Lines indicate mean and shaded regions indicate SD. One of n = 2 biological replicates. (D) Representative images of γH2AX staining after a 45-min incubation in DMSO, ATR inhibitor, or aphidicolin (1 μM). Scale bar, 20 μm. (E) Boxplots showing quantification of γH2AX signal in cycling or QR cells treated with DMSO, ATR inhibitor, or aphidicolin (1 μM). Only cells in S phase at the time of drug addition were included in the analysis, as determined by APC/C^Cdh1^ inactivation. Cycling cells were maintained in full growth media for the duration of the experiment; QR cells were stimulated with growth media 17 hr prior to drug addition. Drugs were added 45 min prior to fixation and staining (n = 449, cycling DMSO; n = 703, QR DMSO; n = 937, QR ATRi; n = 1,377, QR aphidicolin). One of n = 2 biological replicates. (F) S-phase duration in cycling and QR cells as measured by EdU incorporation. Cells were binned by their time since APC/C^Cdh1^ inactivation and median EdU intensity ± SEM was calculated for each bin (n > 50 cells for each bin). EdU values were normalized so that the area under the curve was the same for both conditions. One of n = 2 biological replicates. (G) Model of how stochastic changes in the degree of replication stress cause fluctuating levels of CDK2 activity in a cell. Increases and decreases in CDK2 activity are paralleled by modulations in the rate of DNA replication. See also Figure S5.

Differences in DNA damage between continuously cycling cells and cells re-entering the cell cycle from quiescence have previously been reported (Beerman et al., 2014; Walter et al., 2015). We hypothesized that cells exiting quiescence may also incur increased levels of replication stress and therefore different CDK2 fluctuations and different levels of CDK2 suppression. Quiescence was induced by mitogen starvation for 48 hr followed by serum addition and re-entry into the cell cycle. We observed stereotypical differences in CDK2 activity during S phase in quiescence-release cells compared with continuously cycling cells. Both populations of cells build up CDK2 activity initially in G1 phase. However, shortly following S-phase entry, cells released from quiescence exhibit a transient plateau in averaged CDK2 activity for approximately 10 hr before eventually increasing CDK2 activity again (Figure 5A). This differs from a steadier increase in averaged CDK2 activity throughout S phase in continuously cycling cells. Markedly, we also observed increased S-phase fluctuations in CDK2 activity in cells released from quiescence compared with cycling cells (Figure 5B). To further compare CDK2 fluctuations between cycling cells and cells exiting quiescence, we performed autocorrelation analysis of CDK2 traces. Autocorrelation analysis allows the frequency of otherwise noisy data to be determined. Cycling cells exhibited maximum anticorrelation at a lag of approximately 1.2 hr, compared with less than 1 hr in quiescence-release cells (Figure 5C). Taken together, these results suggest that CDK2 activity fluctuations occur more frequently and more rapidly in cells exiting quiescence than in continuously cycling cells.

Given the increased volatility of CDK2 activity in cells exiting quiescence, we next investigated whether cells exiting quiescence showed evidence of increased replication stress. Histone H2AX is a downstream target of ATR signaling that is phosphorylated in response to replication stress to generate γH2AX (Ward and Chen, 2001). We measured the levels of γH2AX in individual, unperturbed S-phase cells and found a nearly 2-fold greater level of γH2AX in cells that had been released from quiescence by mitogen stimulation (Figures 5D and 5E). As expected, γH2AX staining decreased significantly when ATR was inhibited, and levels were elevated in cells following an aphidicolin pulse (Figure 5E). Accumulation of γH2AX occurred upon S-phase entry during quiescence release, as no γH2AX signal was detected in cells maintained in quiescence or in cells that had not yet entered S phase upon mitogen stimulation (Figure S5A). We observed similar results in BJ-5ta cells when quiescence was induced by mitogen starvation but not when quiescence was induced by contact inhibition, suggesting that increased γH2AX accumulation and replication stress upon quiescence release is unique to growth factor deprivation-induced quiescence (Figure S5B). These results are consistent with previous reports demonstrating unique programs of quiescence depending on the quiescence-inducing stimulus (Coller et al., 2006). Taken together, our results demonstrate that, upon entry into the first S phase out of mitogen starvation, cells encounter increased endogenous replication stress that results in increased ATR-mediated transient repression and increased overall fluctuations in CDK2 activity.

Since CDK2 activity controls the rate of DNA synthesis, we sought to determine how differences in replication stress and CDK2 activity during S phase between cycling and quiescence-release cells affect S-phase progression. In cycling cells, we observed a total S-phase length of approximately 6 hr (Figure 5F). In cells released from quiescence, S-phase duration was approximately 9 hr from APC/C^Cdh1^ inactivation. Notably, quiescence-release cells did not begin to accumulate detectable levels of EdU until approximately 1 hr after APC/C^Cdh1^ inactivation, suggesting that S phase does not begin immediately after the APC/C^Cdh1^ G1 restriction point under these conditions. These results suggest that cells exiting quiescence experience increased replication stress upon entering S phase, which results in greater ATR-mediated repression of CDK2 activity and slower progression through S phase due to decreased rates of DNA synthesis.

## DISCUSSION

Our study shows that the activity of CDK2 fluctuates during an unperturbed S phase, with frequently observed transient decreases and plateaus. Transient repression of CDK2 activity can be artificially induced by brief exogenous addition of replication stress to increase ATR signaling, resulting in rapid CDK2 inhibition that is relieved approximately 40 min after aphidicolin removal. While the major DNA damage-responsive kinases (ATM, ATR, and DNA-PK) are all involved in responding to DNA lesions and replication stress responses (Bensimon et al., 2011), we show that ATR is the primary kinase responsible for inhibiting CDK2 in an unperturbed S phase. We further show that endogenous replication stress causes ATR-mediated fluctuations in CDK2 activity and also controls the overall rate at which CDK2 activity increases during S phase (Figure 5G). We demonstrate that these naturally occurring fluctuations in CDK2 activity dynamically control DNA synthesis, likely as a result of corresponding changes in the rate of firing of origins of replication (Beck et al., 2012; Petermann et al., 2010). This feedback control allows the global rate of replication to be adjusted to resolve elevated replication stress. Finally, we found that cells entering their first S phase following mitogen stimulation exhibit increased ATR-mediated CDK2 fluctuations due to elevated levels of replication stress. Overall, our results reveal a dynamic control principle, showing a link from normal stochastic replication stress to transiently reduced DNA replication rates and reduced speed of S-phase progression mediated by ATR-triggered global transient repression of CDK2 activity.

Our findings provide mechanistic insights into the prior observation that DNA damage during S phase causes a graded slowing of S-phase progression (Chao et al., 2017). While global changes in CDK2 activity and corresponding changes in origin firing have been observed following exogenous addition of genotoxic stress (Yekezare et al., 2013), our study shows that variable endogenous replication stress already results in transient global suppression of CDK2 activity and globally decreased rates of DNA synthesis. It is important to note that we measured global changes in DNA replication and that our assay does not resolve the impact that ATR and CDK2 activity may have on the firing of local origins near sites of DNA replication stress; indeed, the firing of local dormant origins may be promoted near the sites of replication stress (Yekezare et al., 2013). Nevertheless, our strategy may also be more broadly relevant to investigate stress signaling beyond DNA damage. For example, limitation of essential components of DNA replication, such as RPA and dNTPs, have also been shown to cause replication stress (Beck et al., 2012; Toledo et al., 2013). Therefore, the temporary slowing of DNA replication rates via transient ATR-mediated global CDK2 inhibition we discovered may function as an important fast-acting negative feedback mechanism by which cells prevent the demand for nucleotides or other resources from exceeding current cellular supply.

We also note that the scope of our study was limited to examining differences in replication stress and CDK2 activity between two different cell states: continuously cycling and exiting quiescence upon mitogen stimulation. Furthermore, we focused on measuring CDK2 fluctuations in the immortalized but non-transformed breast epithelial cell line, MCF10A. However, the degree of replication stress is known to be highly variably across different cell types and disease states. For example, defects in replication stress response proteins result in a predisposition to a variety of diseases, including premature aging, neurological dysfunction, and cancer (Zeman and Cimprich, 2014). A number of strategies are currently being validated in clinical trials to use DNA damage repair and replication stress response inhibitors in cancer therapy (O’Connor, 2015). Our method for live-cell analysis of fluctuating CDK2 activities and measurements of CDK2 repression in S phase may reveal the degree of replication stress for different cells and conditions. Such studies will contribute to a comprehensive understanding of how DNA damage repair pathway drugs alter CDK2 activity and DNA replication rates. Thus, we believe that our approach has broad methodological value for future studies of replication stress responses in fields such as aging and cancer biology.

## STAR★METHODS

### CONTACT FOR REAGENT AND RESOURCE SHARING

Further information and requests for resources and reagents should be directed to and will be fulfilled by the Lead Contact, Tobias Meyer (tobias1@stanford.edu).

### EXPERIMENTAL MODEL AND SUBJECT DETAILS

#### Cell Lines

Cells were grown in 37°C and 5% CO_2_ and between 30% and 80% confluency. Cells were provided fresh media at least once every three days. Dulbecco Modified Eagle medium (DMEM) and DMEM/F12 were acquired from ThermoFisher Scientific. Cells have not been authenticated but were acquired directly from ATCC.

MCF-10A (ATCC, #CRL-10317, RRID:CVCL_0598, human female) were cultured in phenol red-free DMEM/F12 supplemented with 5% horse serum, 20 ng/mL EGF, 10 mg/mL insulin, 500 mg/mL hydrocortisone, and 100 ng/mL cholera toxin. Starvation media consisted of the same growth media but instead of horse serum, insulin, and EGF, 0.3% BSA is added. Re-suspension media (used to inactivate trypsin) consisted of DMEM/F12 and 20% horse serum (protocol from the laboratory of Joan Brugge).

BJ-5ta (ATCC, #CRL-4001, human male) were cultured in DMEM plus 10% FBS, 20% Medium 199 (ThermoFisher), and 0.02mg/ml hygromycin B. Starvation media consisted of DMEM, 0.1% BSA, and 0.02mg/ml hygromycin B.

### METHOD DETAILS

#### General Experimental Setup and Analysis

Cells are seeded to ensure 30% to 90% confluency throughout the experiment in 96 well plates. For quiescence experiments, MCF-10A cells were washed three times to remove residual growth media then incubated for 48 hr in starvation media prior to mitogen release. To contact inhibit cells, 1 million BJ-5ta cells were plated into 6-well polystyrene plates (we note that different materials result in different cellular adhesion strength and thus affect contact inhibition). The cells were then kept in the dish for 72 hr while growth media is refreshed daily. To release the cells from contact inhibition, cells were trypsinized, seeded onto a 96 well plate, and imaged starting 5 hr after plating.

All constructs were introduced into cells by lentiviral transduction. CSII-pEF-H2B-mTurquoise, CSII-pEF-DHB(aa994–1087)-mVenus, and CSII-pEF-Geminin(aa1–110)-mCherry were described previously (Cappell et al., 2016; Hahn et al., 2009; Spencer et al., 2013). Transduced cells were sorted on a Becton Dickinson Influx to obtain populations expressing the desired fluorescent reporters.

Cells were plated ~20 hr prior to imaging in a 96-well dish (Costar #3904) at 8,000 cells/well. Prior to imaging, 200 mL fresh growth media was added. Time-lapse images were acquired in a humidified, 37°C chamber on an IXμ microscope (Molecular Devices) with a 10X 0.3NA objective. Images were acquired every 12 min in the CFP, YFP, and TexasRed channels, and total exposure time was kept under 500 ms for each time point. Cells were tracked using custom MATLAB scripts.

The drugs used in this study were: aphidicolin (125 – 1000 nM, Cayman Chemical, #14007), neocarzinostatin (100–200 ng/ml, Sigma-Aldrich, N9162), AZ-20 (2 μM, ATR inhibitor, Cayman Chemical, #17589), Ku-60019 (5 μM, ATM inhibitor, Cayman Chemical, #17502), NU-7441 (1 μM, DNA-PK inhibitor, Cayman Chemical, #14881), Chir-124 (250 nM, Chk1 inhibitor, Cayman Chemical, #16553), MK-1775 (1 μM, Wee1 inhibitor, Cayman Chemical, #21266), CDK2 inhibitor III (60 μM, EMD Millipore, #238803), RO3306 (3, 10 μM, Cdk1 inhibitor, Sigma-Aldrich SML0569), hydroxyurea (100, 1000 mM, Sigma Aldrich #H8627).

Cells were fixed in 4% paraformaldehyde, washed three times with PBS, permeabilized in 0.1% triton, blocked in 1% BSA, 10% FBS, and 0.01% NaN_3_ then stained overnight at 4°C with primary antibody. Primary antibodies used in this study were anti-phospho-H2AX (Cell Signaling Technologies, #9718) and anti-BrdU (BD Biosciences, #347580). Visualization was performed using a secondary antibody conjugated to AF-568 or AF-647 and imaged with TexasRed and Cy5 filter cubes, respectively. EdU staining was performed by adding 10 μM EdU to cells for 15 min prior to washout or fixation, and visualization was performed according to manufacturer’s instructions (Invitrogen, #C10356).

For EdU/BrdU co-labeling experiment, cycling cells were pre-imaged for 12 hr, pulsed with 10 μM EdU for 10 min, washed three times and replenished with fresh growth media, imaged for 50 min, pulsed with 100 μM BrdU and imaged for 10 min then fixed in 4% paraformaldehyde. Following permeabilization and blocking as above, EdU was visualized according to manufacturer’s instructions (Invitrogen, #C10356). BrdU was visualized following EdU visualization by treating cells with 1.5N HCl for 30 mins, followed by incubation overnight at 4°C with anti-BrdU primary antibody (BD Biosciences, #347580). Change in nucleotide incorporation was calculated by first gating for S-phase cells (EdU- and BrdU-positive). Single-cell EdU incorporation rate was calculated by measuring the EdU intensity in a cell and determining the percentile of that intensity within the population intensity of all EdU-positive cells. BrdU was calculated similarly. Change in nucleotide incorporation rate was then calculated as BrdU_rate_ – EdU_rate_ in each cell. Change in CDK2 activity in each cell was determined as CDK2 activity at the time of the BrdU pulse minus CDK2 activity at the time of the EdU pulse.

Information about replicates and error bars can be found in the figure legends. Automated-pipeline scripts performed all analyses. In analyses where only certain populations of cells are analyzed (for example, G0/G1 cells), the gating criteria are described in the figures, figure legends, or results section. Experiments are excluded when cells are of suboptimal confluency and/or stressed (<30% of control cells in S/G2 24hrs after mitogen release or when asynchronously cycling). Individual cells with low or absent DHB-Venus expression (bottom 5% of cells) are removed. Noisy traces, defined as those that had a change in the cytoplasmic/nuclear ratio of DHB-Venus greater than 0.25 between two subsequent time points, are also removed from further analysis.

#### Computational Modeling

The model calculations describing CDK2 fluctuations were based on the assumption that CDK2 activity linearly increases in S phase (it is also assumed to be linear in G2 and equal 0 in G0/early G1). This assumption is based on the linear increase in CDK2 activity in S phase seen in experiments where we inhibited wither ATR or Wee1. We then assumed that 20 stochastic replication stress events may occur during S phase (at time = t_i_) with the same amplitude (α_i_), generating an approximately 1 hour long proportional dip in the CDK2 activity. We modeled this transient repression of CDK2 activity as 20 normally distributed added CDK2 dip functions. The function shown is: CDK2(t)=CDK2_linear_(t) – Σ_1:20_ α*exp(−(t-t_i_)^2^/.5).

### QUANTIFICATION AND STATISTICAL ANALYSIS

#### Image and Data Analysis

Segmentation was performed using either Hoechst (fixed-cell) or H2B-CFP (live-cell), as described previously (Cappell et al., 2016). For DHB-Ven measurements, a perinuclear ring with an inner radius 2 μm outside the nuclear mask and an outer radius 10 μm outside the nuclear mask was used for cytoplasmic measurements. Regions of this ring within 10 μm of another nucleus were excluded. Only regions of the ring above background were included for further analysis. Global background subtraction was performed for each channel. To calculate background intensity, nuclear masks were dilated by 50 μm and the median pixel intensity of all non-masked regions was calculated. Nuclear fluorescence intensity of DHB-Venus, Geminin-mCherry, BrdU, and EdU was calculated as the median nuclear foreground intensity. DHB-Venus cytoplasmic intensity was calculated as the 75^th^ percentile of the foreground of the perinuclear ring. DHB-Venus translocation, our measure of CDK2 activity, was calculated as the ratio of the cytoplasmic signal over the nuclear signal. Nuclear γH2AX signal was calculated by generating a foreground mask of γH2AX puncta via top hat filtering the raw image with a circular kernel of radius 4 μm and thresholding on absolute intensity. γH2AX puncta intensity was calculated as the total number of pixels above background in each nuclear region. Tracking of cells between time-lapse images and calling the time of CDK2 and geminin-fragment rise have been previously described (Cappell et al., 2016). DHB-Venus traces were smoothed using a moving window of three time points for all experiments.

To quantify CDK2 fluctuations, cells were computationally aligned to the time of CDK2 rise. A best-fit curve for each CDK2 trace was generated using a third-degree polynomial function. The difference squared between the CDK2 activity trace and the polynomial function was calculated for each time point. The difference squared value was reported as a measure of CDK2 fluctuations. To generate mean traces, the mean value and standard error of the mean of the squared difference were calculated for each time point after CDK2 rise in the population of cells.

#### Statistical Analysis

Time-series data were compared at each timepoint by a Mann-Whitney U test and corrected for multiple comparisons by the Bonferroni method. Column data were compared using a t-test (if normally distributed) or a Mann-Whitney U test (if not normally distributed). Multiple groups were compared using a one-way ANOVA and Tukey’s multiple comparisons test (if normally distributed) or a Kruskall-Wallis and Dunn’s multiple comparisons test (if not normally distributed). For the box plots, the box ends are the quartiles, the horizontal line inside the box is the median, and the whiskers extend out to the farthest points that are not outliers. Further statistical details of experiments can be found in the figure legends.

### DATA AND SOFTWARE AVAILABILITY

Further information and all reasonable data and code requests can be fulfilled by the Lead Contact, Tobias Meyer (tobias1@stanford.edu).

## Supplementary Material

Supplementary Materials

Supplementary Video

## Figures and Tables

**Table:** KEY RESOURCES TABLE

REAGENT or RESOURCE	SOURCE	IDENTIFIER
Antibodies		
Rabbit polyclonal anti-phospho-γH2A.X(S139)	Cell Signaling	Cat#2577; Lot 11; RRID: AB_2118010
Mouse BrdU antibody	BD Biosciences	Cat#347580; Clone B44; Lot: 6042756; RRID: AB_400326
Chemicals, Peptides, and Reoombinant Proteins		
CDK1 inhibitor RO3306	Sigma-Aldrich	Cat#SML0569; CAS 872573-93-8
CDK2 inhibitor III	EMD Millipore	Cat#238803; CAS 199986-75-9
Aphidicolin	Cayman Chemical	Cat#14007; CAS 38966-21-1
Hydroxyurea	Sigma Aldrich	Cat#H8627; CAS 127-07-1
Neocarzinostatin	Sigma-Aldrich	Cat#N9162; CAS 9014-02-2
Ku-60019 (ATMi)	Cayman Chemical	Cat#17502; CAS 925701-46-8
NU-7441 (DNA-PKi)	Cayman Chemical	Cat#14881; CAS 503468-05-9
Chir-124 (Chk1i)	Cayman Chemical	Cat#16553; CAS 405168-58-3
MK-1775 (Wee1i)	Cayman Chemical	Cat#21266; CAS 955365-80-7
AZ-20 (ATRi)	Cayman Chemical	Cat#17589; CAS 1233339-22-4
Critical Commercial Assays		
Click-iT EdU Alexa Fluor 647 Imaging Kit	ThermoFisher Scientific	Cat#10340
Experimental Models: Cell Lines		
Human: MCF-10A	ATCC	ATCC #CRL-10317, RRID:CVCL_0598
Human: BJ-5ta	ATCC	ATCC #CRL-4001
Recombinant DNA		
CSII-pEF-H2B-mTurquoise	Spencer et al., 2013	N/A
CSII-pEF-DHB(aa994–1087)-mVenus	Hahn et al., 2009	N/A
CSII-pEF-Geminin(aa1–110)-mCherry	Sakaue-Sawano et al., 2008.	N/A
Software and Algorithms		
Cell tracking and cell analysis	Cappell et al., 2016	N/A

## Highlights

- Live single-cell analysis reveals fluctuating CDK2 activity in S phase
- Fluctuating CDK2 activity is caused by endogenous replication stress
- CDK2 activity fluctuates to adjust global DNA synthesis rate
- Serum-starved cells exit quiescence with more replication stress and longer S phase
