# Supplementary material for: Stochastic Endogenous Replication Stress Causes ATR-Triggered Fluctuations in CDK2 Activity that Dynamically Adjust Global DNA Synthesis Rates
Source: Cell Syst. Author manuscript; Available in PMC 2019 Mar 27. (PMC6436092; doi:10.1016/j.cels.2018.05.011)
Supplement: Supplementary Materials [file NIHMS1015315-supplement-Supplementary_Materials.pdf]

Cell Systems, Volume 7

Supplemental Information

Stochastic Endogenous Replication Stress Causes ATR-Triggered Fluctuations in CDK2 Activity that Dynamically Adjust Global DNA Synthesis Rates

Leighton H. Daigh, Chad Liu, Mingyu Chung, Karlene A. Cimprich, and Tobias Meyer

Figure S1

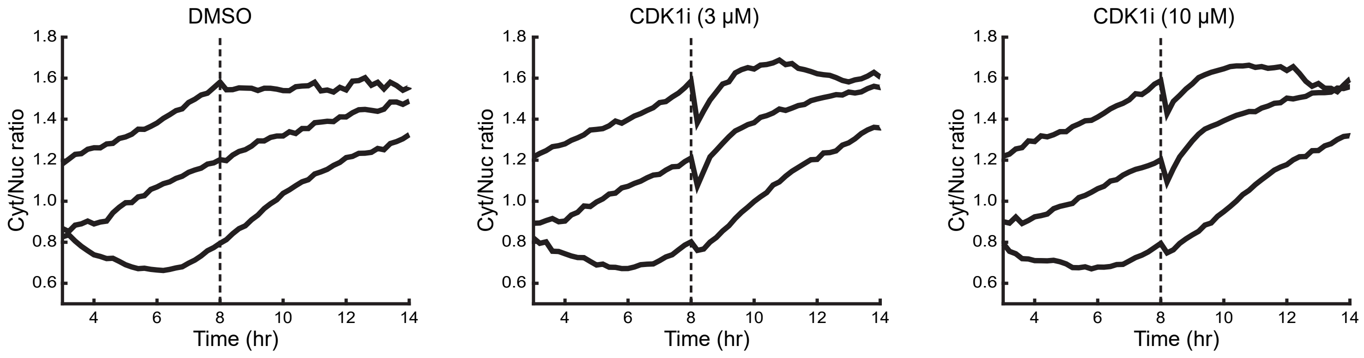

Figure S1. Further characterization of DHB-Ven specificity in MCF10A cells, Related to Figure 1

Median traces of DHB-Ven cytoplasmic:nuclear ratio in cycling MCF10A cells treated with DMSO or different doses of a specific CDK1 inhibitor. Cells were computationally gated for cells at various levels of DHB-Ven cytoplasmic:nuclear ratio at the time of drug addition (n > 500 cells for each condition). Dashed line indicates the time of drug addition. Drugs remained in the media for the duration of the experiment.

Figure S2

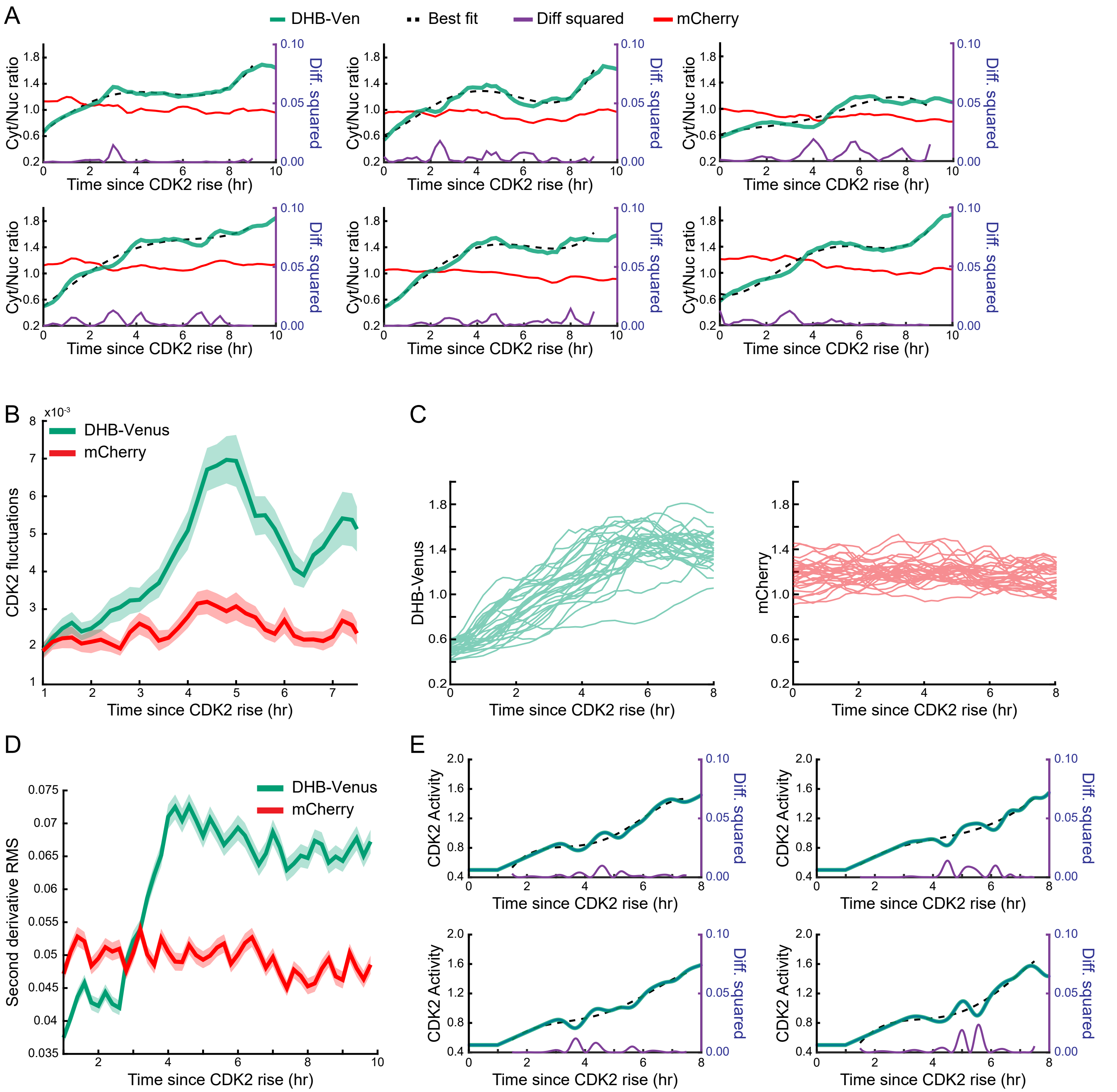
A
DHB-Ven
Best fit
Diff squared
mCherry
Cyt/Nuc ratio
Diff. squared
Time since CDK2 rise (hr)
B
x10-3
DHB-Venus
mCherry
CDK2 fluctuations
Time since CDK2 rise (hr)
C
DHB-Venus
mCherry
Time since CDK2 rise (hr)
D
Second derivative RMS
DHB-Venus
mCherry
Time since CDK2 rise (hr)
E
CDK2 Activity
Diff. squared
Time since CDK2 rise (hr)

Figure S2. CDK2 fluctuations are not due to measurement noise, Related to Figure 1

(A) Single-cell traces beginning at the time of CDK2 activity rise. Cells expressed both DHB-Venus and mCherry. The cytoplasmic:nuclear ratio of DHB-Venus (green line; measure of CDK2 activity) and an unconjugated mCherry (red line; control) are shown for each cell. A polynomial fit for each DHB-Venus trace (black, dashed line) and the squared difference at each time-point between the single-cell DHB-Venus trace and the polynomial fit line (blue line) are shown.
(B) Measurement of CDK2 fluctuations in single cells expressing the DHB-Ven CDK2 reporter and an untagged mCherry control following release from quiescence. Gating was performed to include only cells where the average mCherry cytoplasmic:nuclear ratio was greater than 1.1 over the course of imaging. Cells were computationally aligned to the time of initial CDK2 activity rise. Fluctuations were quantified by determining the difference squared between individual traces and a polynomial fit curve. Lines indicate mean trace and shaded regions indicate SEM (n = 200 cells).
(C) Example single-cell traces from (B) of DHB-Venus cytoplasmic:nuclear ratio and the corresponding mCherry cytoplasmic:nuclear ratios, demonstrating that cytoplasmic:nuclear ratios were similar between DHB-Venus and mCherry when calculating CDK2 fluctuations (n = 30 cells).
(D) Alternative method of measuring CDK2 fluctuations using the root-mean-square (RMS) of the second derivative of single-cell traces from Figure 1G. Cells were computationally aligned to the time of CDK2 rise. Lines indicate mean trace and shaded regions indicate SEM (n = 560).
(E) Polynomial fit accurately detects fluctuations in computationally simulated CDK2 traces.

Figure S3

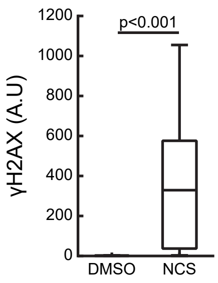

Figure S3. Neocarzinostatin induces DNA damage in MCF10A cells, Related to Figure 2

Cycling MCF10A cells were treated with 800ng/ml neocarzinostatin (NCS) for 16 hours prior to fixation and staining for γH2AX (n = 26344, DMSO; n = 12118, NCS).

Figure S4

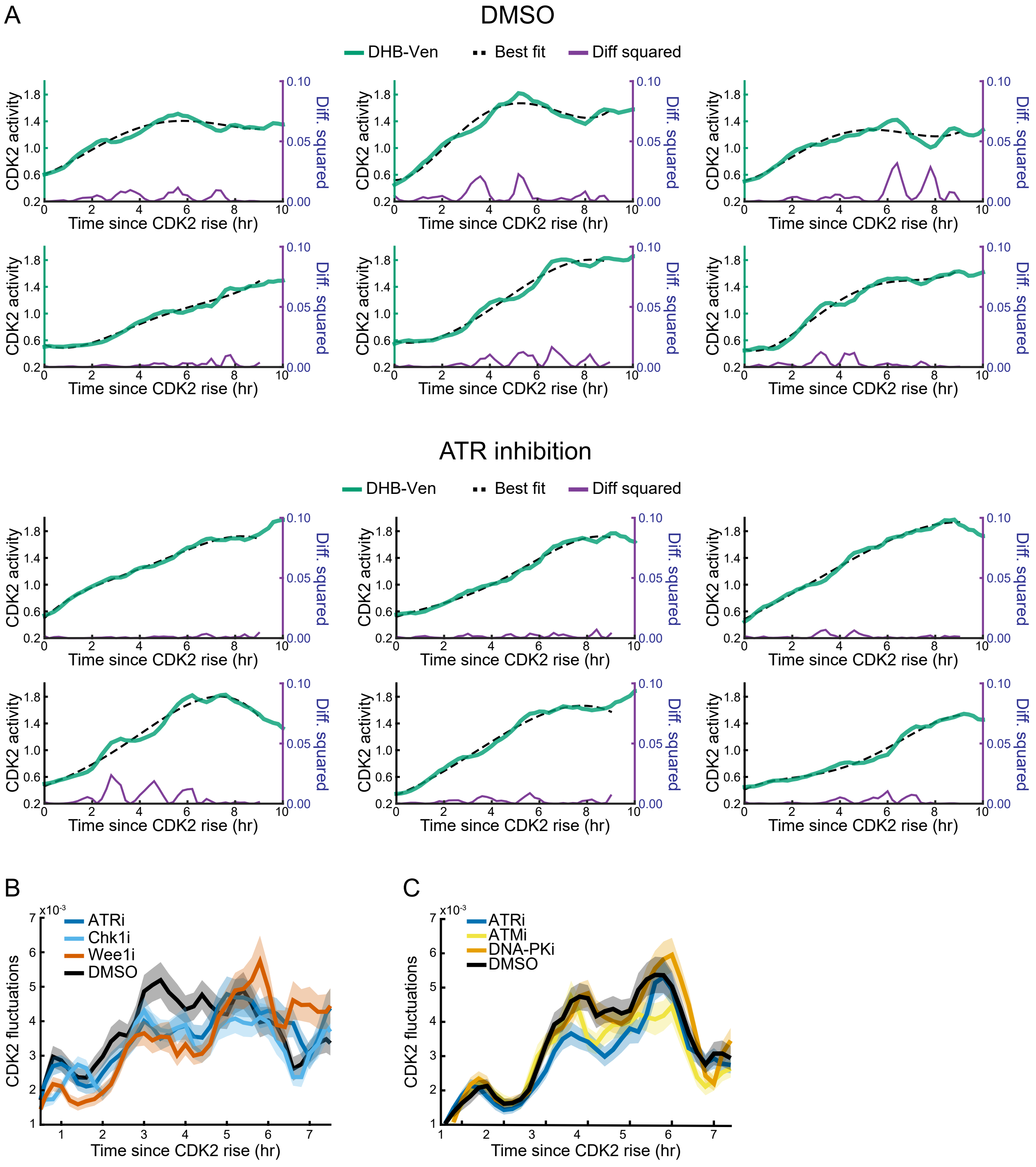
A
DMSO
DHB-Ven
Best fit
Diff squared
CDK2 activity
Diff. squared
Time since CDK2 rise (hr)
ATR inhibition
B
ATRi
Chk1i
Wee1i
DMSO
CDK2 fluctuations
x10-3
C
ATRi
ATMi
DNA-PKi
DMSO

Figure S4. ATR pathway inhibition increases S-phase CDK2 activity and decreases S-phase CDK2 fluctuations, Related to Figure 3

(A) Example single-cell traces of cells stimulated to exit quiescence by mitogen addition and treated with either DMSO or ATR inhibitor eight hours after mitogen stimulation, prior to CDK2 rise. Traces are shown beginning at the time of CDK2 activity rise. For each cell, CDK2 activity (green line), a polynomial fit curve to the CDK2 activity trace (dashed black line) and the squared difference between the CDK2 activity trace and the polynomial fit curve (blue line) are shown.
(B) Fluctuation analysis of CDK2 activity in cells treated with DMSO, ATR inhibitor, Chk1 inhibitor, or Wee1 inhibitor prior to S-phase and computationally-aligned to the time of CDK2 activity rise. Lines indicate mean trace and shaded regions indicate SEM (n = 220 for all conditions). 1 of n=2 biological replicates.
(C) Fluctuation analysis of CDK2 activity in cells treated with DMSO, ATM inhibitor, ATR inhibitor, or DNA-PK inhibitor prior to S phase and computationally-aligned to the time of CDK2 activity rise. Lines indicate mean trace and shaded regions indicate SEM (n = 220 for all conditions). 1 of n=2 biological replicates.

Figure S5

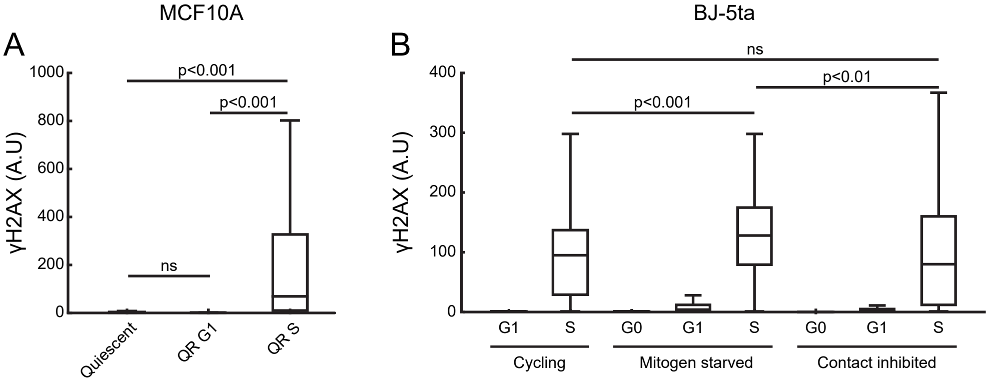

Figure S5. Cells released from mitogen starvation incur increased DNA damage upon S-phase entry, Related to Figure 5

(A) Boxplots showing quantification of γH2AX signal in quiescent or quiescence-release (QR) MCF10A cells. Quiescence-release cells were stimulated with mitogens 20 hours before fixation and staining. Quiescent cells were maintained in starvation media for the duration of the experiment. S-phase cells were determined by EdU incorporation (n =8101, quiescent; n = 9787, QR G1 phase; n = 8682, QR S phase).

(B) Boxplots showing quantification of γH2AX signal in BJ-5ta cells under cycling or quiescence release (QR) conditions. Cycling cells were maintained in full growth media for the duration of the experiment (n =605, G1; n =248, S phase). Mitogen-starved cells were deprived of growth factors for 48 hours to induce quiescence then either maintained in starvation media (quiescent cells, G0) or stimulated for 24.5 hours prior to fixation and staining (n =2440, G0/quiescent; n =74, QR G1 phase; n =155, QR S phase). Contact inhibited cells were maintained at confluence for three days in full growth media to induce quiescence. G0, quiescent cells were maintained at confluence prior to fixation and imaging. Quiescence-release cells were trypsinized and re-plated at a lower density 24.5 hours prior to fixation and staining (n =670, G0/quiescent; n = 483, QR G1 phase; n =385, QR S phase). S-phase cells were determined by EdU incorporation.
